# Parasite-associated microbiomes in *Ascaris* and *Trichuris* nematodes: current evidence, challenges, and future directions

**DOI:** 10.1128/msystems.00569-26

**Published:** 2026-09-23

**Authors:** Shezryna Shahrizal, Mohd Shafiq Aazmi, Yvonne Ai Lian Lim, Azdayanti Muslim

**Affiliations:** 1 Department of Medical Microbiology and Parasitology, Faculty of Medicine, Universiti Teknologi MARA, Cawangan Selangor, Kampus Sungai Buloh 54703, Sungai Buloh, Selangor, Malaysia; 2 Institute of Medical Molecular Biotechnology, Universiti Teknologi MARA, Cawangan Selangor, Kampus Sungai Buloh 54703, Sungai Buloh, Selangor, Malaysia; 3 School of Biology, Faculty of Applied Science, Universiti Teknologi MARA 54703, Shah Alam, Selangor, Malaysia; 4 Microbiome Health and Environment (MiHeaRT), Faculty of Applied Sciences, Universiti Teknologi MARA 54703, Shah Alam, Selangor, Malaysia; 5 Department of Parasitology, Faculty of Medicine, Universiti Malaya 37447, Kuala Lumpur, Malaysia; 6 Institute for Biodiversity and Sustainable Development, Universiti Teknologi MARA 54703, Shah Alam, Selangor, Malaysia; University of California, San Francisco, San Francisco, California, USA

**Keywords:** parasite-associated microbiomes, *Ascaris*, *Trichuris*, gut microbiota

## Abstract

Soil-transmitted helminth (STH) parasites, particularly *Ascaris lumbricoides* and *Trichuris trichiura*, co-exist and co-evolve with a complex human microbiota within the gut ecosystem. Most research has focused on how parasitic nematodes affect the host gut microbial structure and its consequences. In contrast, studies on the parasites’ own associated microbiota remain underexplored. Understanding this gap is crucial, as it adds another layer of complexity to holobiont-holobiont interactions. Driven by the Parasite Microbiome Project, this review summarizes current evidence on *Ascaris-* and *Trichuris*-associated microbiomes from both natural and experimental studies, with a focus on their compositions, potential functional roles, challenges, and future directions. Existing findings are largely based on microbiomes associated with the gut of the parasites and are often characterized by transient, host-derived communities acquired through horizontal transmission, which may contribute to parasite survival and host interaction. We therefore emphasize the need to expand investigations to other specific tissues, particularly reproductive organs, as potential sites of a stable and conserved microbiota in STH parasites. Understanding this aspect is essential for advancing our knowledge of parasite-microbiome-host interactions in STH infections and may uncover the potential of microbiome-based therapeutic interventions for STH control and prevention.

## INTRODUCTION

Soil-transmitted helminths (STHs) refer to a group of parasitic nematode worms that infect humans and are transmitted primarily via soil contaminated with eggs or larvae passed in the feces of infected individuals. The STH species include *Ascaris lumbricoides* (roundworm), *Trichuris trichiura* (whipworm), *Ancylostoma duodenale*, and *Necator americanus* (hookworms), and *Strongyloides stercoralis*. Globally, these infections remain among the most prevalent neglected tropical diseases, affecting an estimated 1.5 billion people, or ~24% of the world’s population, predominantly in low- and middle-income countries (1). Among these, *A. lumbricoides* and *T. trichiura* contribute substantially to the global burden, with an estimated 772–892 million and 429–508 million infections, respectively (1, 2).

These parasites inhabit the gastrointestinal tract of humans, and in many cases, infections are asymptomatic, particularly when parasite burden is low. However, when symptoms do occur, they commonly include abdominal discomfort and diarrhea. Although STH infections rarely cause direct mortality, they represent a major source of morbidity, accounting for millions of disability-adjusted life years (DALYs) lost worldwide. Recent estimates based on the Global Burden of Diseases study indicate that approximately 1.38 million DALYs were attributable to STH infections globally in 2021, with ascariasis contributing the largest proportion of this burden with 293.80 million cases and 647.53 thousand DALYs (3). Chronic infections or severe burden are most frequently observed in pre-school and school-aged children, which can lead to significant health problems, including malnutrition, anemia, and impaired growth and cognitive development (4, 5). In STH-endemic populations where adequate sanitation is lacking, reinfection remains common despite mass drug administration, highlighting the long-term persistence and co-existence of these parasites within the human gut. These infections also contribute to broader social and economic burdens, including reduced productivity and overall well-being (1).

As these helminths inhabit the same ecological niche as the gut bacteria, advances in high-throughput sequencing over the past 15 years have facilitated studies on the interactions between STH infections and the host gut microbiota. Most findings suggest that the infections are closely linked to alterations in microbial diversity, taxonomic composition, and metabolic function of the host gut microbiota (6–9). This, in turn, results in changes in the balance of beneficial and harmful microbiota within the gut (9, 10). This reflects how most organisms, including humans, must adapt to survive when entering a new environment. Theoretically, helminths inhabiting the gut system must also strive to establish and survive in a complex microbial ecosystem by not only evading host immune responses but also adapting to the surrounding microbiota and available resources. Hence, helminths appear to modify the gut environment as part of their survival strategy, modulating host microbiota to promote conditions favorable for their establishment, egg hatching, and even immune evasion (11–13). These findings have shaped current understanding of helminth-microbiota-host interactions, particularly in modulating host immunity indirectly via microbiome-mediated mechanisms (12–15).

Nevertheless, STHs are not sterile entities. Instead, the parasites themselves harbor their own associated microbial communities (16). It is believed that each species has different bacterial taxa shaped by the biology of the parasite and its environment. These bacteria may contribute to parasite fitness, supporting lifecycle transitions and resilience under environmental stress, adding another layer of complexity beyond the parasite infection-host microbiota alone (17). A well-studied example is the mutualistic relationship between the filarial worm *Brugia malayi* and its endosymbiont *Wolbachia*, where the bacteria provide essential cofactors such as riboflavin and nucleotides in exchange for amino acids as part of the development and survival strategies of the parasite (18–20). Free-living nematodes such as *Acrobeloides maximus* and *Caenorhabditis elegans* also harbor species-associated microbiota that differ from the surrounding soil environment, implying selective acquisition or maintenance of bacterial communities (21, 22). Dominant taxa found in these nematodes include Enterobacteriaceae, Pseudomonadaceae, and *Ochrobactrum*, some of which have roles in nutrition, thermotolerance (the ability to survive high temperatures), and pathogen defense or resistance to oxidative stress (21–23). Trematodes such as *Coitocaecum parvum* have also been shown to harbor a phylogenetically diverse microbiota that is distinct from its hosts and surrounding environment (24). The same study also demonstrated that a small core set of bacterial taxa is maintained across all life stages, suggesting potential vertical transmission and functional roles. All these observations support the holobiont theory (25), in which a host and its associated microbiota are considered as a single biological entity. Building on this concept, Dheilly highlighted that host-parasite relationships may be viewed as holobiont-holobiont interactions (26), where both the host and the parasite interact together with their respective microbial communities to drive the development and evolution of disease.

However, current understanding of microbiomes residing within the geohelminth parasites, such as *Ascaris* and *Trichuris*, remains underexplored, despite extensive studies on host gut microbiota-STH interactions. In addition, Dheilly et al. proposed the Parasite Microbiome Project (PMP), aimed at systematically characterizing parasite-associated microbiota and promoting a more comprehensive exploration of parasitic microbiomes (27). The PMP has provided a conceptual and methodological framework emphasizing rigorous contamination control and challenges in identifying both core and transient microbiomes that contribute to parasite survival or adaptation in the gut environment (27, 28). Driven by that, this review collectively updates existing knowledge on *Ascaris-* and *Trichuris-*associated bacterial microbiomes to increase understanding of STH-associated microbiomes, explore their potential functional roles, and identify major research gaps for future investigation.

## GUT MICROBIOME PROFILE OF THE *ASCARIS* AND *TRICHURIS*

Although the concept of helminths harboring their own associated microbiota (helminth holobiont) is not entirely new and has been proposed in other parasitic organisms, investigations specific to STH parasites are still at infancy, especially in human studies, due to ethical limitations and challenges in parasite recovery. In this paper, we collectively summarize recent studies on *Ascaris-* and *Trichuris*-associated bacteriome based on 16S rRNA sequencing in both experimental and natural infections, as shown in Table 1. Despite differences in geographic regions, parasite species, host types, and study designs, notable similarities were observed, providing important insights into STH-microbiome interactions.

**TABLE 1:** The summaries of the selected *Ascaris* and *Trichuris*-associated microbiota studies

Reference (country)	Parasite species, stage, and sample type	Host/model	16S rRNA target region	Bacterial microbiome of the parasite	Key findings and limitations
White et al. (29) (United Kingdom)	*T. muris* adult (gut) and host gut microbiota	Experimental mice	V1–V3 (454 pyrosequencing)	Phylum Firmicutes (family Lachnospiraceae). Proteobacteria (families Enterobacteriaceae, Oxalobacteraceae, Sphingomonadaceae—notably enriched compared to host gut). Bacteroidetes (families Bacteroidales S24-7 group, Rikenellaceae, Prevotellaceae).	*T. muris* adults harbor a distinct gut microbiota compared to its host and selectively acquire and maintain this microbiota after hatching from the host environment (as the embryonated eggs used were bacterium free), indicating horizontal acquisition rather than vertical transmission (from parent generation). Depletion of the *T. muris* microbiota by antibiotics caused a rapid loss of parasite motility and death. *T. muris* requires host microbiota for survival and development, as germ-free mice failed to support *T. muris* infection and it alters host gut microbiota in regulating parasite burden. Animal model. Extrapolation to human *T. trichiura* infections should be made with caution and requires further validation.
García-Sánchez et al. (30) (Spain)	*T. trichiura* and *T. suis* (whole adult worm, intestine, and reproductive tissues)	Macaque and pig	V3–V4 (Illumina Miseq)	Phyla Proteobacteria, Firmicutes, Actinobacteria, Bacteroidetes, Cyanobacteria. Families Pseudomonadaceae, Methylobacteriaceae, Enterobacteriaceae. Potential pathogenic pathogens detected (*Escherichia*/*Shigella*, *Salmonella*, *Bartonella*, *Mycobacterium*, *Clostridium, Rickettsia, Aeromonas*).	*Trichuris* harbor rich and diverse internal microbiota that are distinct from host gut microbiota. *Trichuris* may act as reservoirs or carriers for pathogenic bacteria. Microbiota composition varies by parasite tissue, sex, and host but remains relatively similar within comparable parasite categories. Small sample size and no direct functional validation of bacterial roles.
Klomkliew et al. (31) (Thailand)	19 *Ascaris lumbricoides* adult (interior and posterior gut), 30 human fecal samples	Human	V4 (Illumina Miseq)	Dominance of Firmicutes (*Streptococcus*, *Lactococcus*) and Proteobacteria. Parasite bacteriome differed from host feces. Infection intensity correlated with parasite gut bacteriome but not host fecal microbiome; metabolome linked to parasite fecundity.	*A. lumbricoides* gut bacteriome is distinct with significantly lower diversity in contrast to the host gut. Anterior and posterior regions of the *A. lumbricoides* gut harbored nearly similar microbiota. Infection intensity strongly influenced the parasite bacteriome, but not the host gut microbiota. *Streptococcus* dominated parasite microbiota in severe infection, whereas *Lactococcus* was prominent in light infections. Heavy infections were associated with metabolites involved in amino acid, lipid, nucleotide, and steroid biosynthesis, which might involve in increased egg production. Cross-sectional study.
Midha et al. (16) (Germany)	*Ascaris suum* adult (gut) and host gut compartment	Experimental pig	V3–V4 (Illumina Miseq)	Phyla Firmicutes (dominant), Proteobacteria, Bacteroidetes, Actinobacteria. Genera *Clostridium*, *Lactobacillus*, *Prevotella*, *Escherichia-Shigella*, Romboutsia, *Streptococcus*.	*A. suum* harbors a distinct and lower diversity of bacteria derived from the host jejunal microbiota. Hatched larvae *in vitro* were bacterium free, suggesting that *A. suum* does not inherit its microbiome and colonization occurs after entering the host. *A. suum* collected from the same host have more similar microbiota to each other than worms from different hosts. Male and female worms show nearly identical microbial structures. Animal model. Interpretation to human *A. lumbricoides* requires caution.
Muslim et al. (32) (Malaysia)	*Ascaris lumbricoides* adult (gut), human fecal samples	Human	V3–V4 (Illumina Miseq)	Phyla Firmicutes (notably *Clostridium*, *Veillonella, Streptococcus*) and Proteobacteria (*Campylobacter*, *Enterococcus*). In contrast, the human host gut microbiota was dominated by Firmicutes and Bacteroidetes.	*A. lumbricoides* harbors a distinct, parasite-specific gut microbiota that is clearly separated from that of its human host. The parasite gut microbiota exhibits significantly lower bacterial diversity and richness compared with human gut microbiota. Preliminary study. Cross-sectional. Small sample size. Single host population.
Castañeda et al. (33) (Colombia)	*Ascaris suum* across all larval stages (L3 and L4), *in vitro*, liver, lung, intestine	Experimental mice	V4 (Illumina NovaSeq)	*A. suum* larva microbiota dominated by Proteobacteria (most abundant and consistently enriched vs host microbiota), Firmicutes, Bacteroidetes, and Actinobacteriota. Enrichment of *Bradyrhizobium*, *Achromobacter*, and *Pseudomonas* during migration.	*A. suum* larvae have a distinct microbiota that is different from host gut, liver, and lung microbiota. Diversity of microbiota increases as larvae migrate through host organs. Early larvae are dominated by Proteobacteria, and later larvae acquire more Firmicutes from the host, suggesting horizontal transmission of microbiome. Partial microbial exchange occurs between larvae and host tissue during migration. Animal model. Larval stages only. Functional roles remain speculative.

The earliest understanding of STH-associated microbiomes was derived from comprehensive experimental research in mice on *Trichuris muris* (29). Do STH parasites harbor their own microbiomes? White et al. compared the bacterial communities in the gut of the adult *T. muris* with the gut bacteria of their murine host. The parasite harbors a distinct gut microbiota, dominated mainly by the phyla Bacteroidetes and Firmicutes, with a significant enrichment of Proteobacteria. *Ex vivo* antibiotic treatment further revealed that depletion of these parasite-associated microbiota significantly reduced parasite fitness, leading to death compared to the control condition, highlighting their important role. In addition, White et al. demonstrated *T. muris* dependency on host microbiota for successful egg hatching and subsequent larval development, with even a single commensal host bacterium being sufficient to support establishment. Overall, White et al. suggested that *T. muris* employs a dual microbiota-mediated strategy by simultaneously shaping its own microbiota and manipulating the host gut microbial environment to establish and survive in the host gut (29).

Subsequent investigations of parasite-associated microbiomes in adult *T. suis* and *T. trichiura* from pigs and macaques (30), *A. lumbricoides* isolated from natural human infections (31, 32), and an experimental study on *A. suum* from pigs (16) consistently demonstrated that these parasites harbor distinct and unique bacterial communities compared to their hosts, despite living within the same gut environment. This finding raises a second question regarding these microbiome acquisitions. Do these helminths simply acquire bacteria from the host they parasitize and the surrounding environment (horizontal transmission), or do they harbor their own indigenous microbiota through vertical transmission? Evidence from both *T. muris* (29) and *A. suum* (16) experimental studies strongly supports horizontal acquisition from the host environment following infection rather than vertical transmission. In the experimental study by White et al., embryonated eggs and newly hatched larvae of *T. muris* were bacterium free or sterile, indicating that the parasite acquires its microbiota after exposure to the host intestine (29). Midha et al. further showed that the *A. suum* microbiome closely resembles the host (pig) jejunal microbiota but is selectively enriched for specific taxa derived from the host (16). A similar trend was observed in *A. lumbricoides* studies, highlighting that only a subset of host-associated bacteria is retained within the parasite gut, suggesting selective acquisition and filtering of host-derived microbes (31, 32). Recent observations in *A. suum* larvae provide further insight by demonstrating that larvae harbor dynamic and stage-specific microbiota, with increasing diversity and potential bacterial exchange during the tissue migration phase (liver, lung, and intestines), suggesting that parasite-associated microbiomes are influenced by parasite life stage and host environment (33).

The next important question is, based on current studies, what are the main bacterial taxa in *Ascaris-* and *Trichuris*-associated microbiomes? Do these parasites share common taxonomic signatures despite differences in species, geographical settings, types of hosts, and study design? Unlike the human gut microbiome, which is primarily dominated by Firmicutes and Bacteroidetes at the phylum level, the *Ascaris-* and *Trichuris*-associated microbiome is predominantly composed of Firmicutes and Proteobacteria but shows heterogeneity at the genus level. In adult *A. lumbricoides* recovered from naturally infected human hosts, parasite gut communities were enriched particularly with *Clostridium*, *Streptococcus*, and *Lactococcus*. These bacteria substantially reduced the representation of Bacteroidetes (highly abundant in human host gut microbiota), indicating that not all host-derived bacteria were selected and may not be compatible with the parasite gut ecosystem (31, 32). Similar enrichment of Firmicutes, but with some representations of Proteobacteria, Bacteroidetes, and Actinobacteria, were also reported in adult *A. suum* from pig models, suggesting the possibility of a host-environment-dependent rather than conserved or core parasitic-specific microbiome (16). Interestingly, the larvae of *A. suum* also exhibited higher bacterial diversity from the phylum Proteobacteria but were enriched with the genera *Bradyrhizobium*, *Achromobacter*, and *Pseudomonas* (33), taxa which are largely absent from adult-stage studies. This further supports the hypothesis of a highly dynamic microbiome with stage-specific dependency shaped by physiological requirements and environments during migration. Similarly, despite showing greater taxonomic diversity, *Trichuris*-associated microbiomes also revealed representation of Proteobacteria such as the genera *Enterobacteriaceae*, *Sphingomonadaceae*, and *Oxalobacteraceae* (29, 30). However, unlike *Trichuris*, which does not undergo tissue migration during its life cycle, the dynamicity and potentially transient microbial associations may be more prominent in *Ascaris* to facilitate adaptation in the human host. Notably, Garcia-Sánchez et al. highlighted the presence of genera of potential opportunistic pathogens, including *Escherichia*/*Shigella* and *Salmonella*, in *Trichuris* (30), raising the possibility that parasites may act as reservoirs or vectors of bacterial pathogens, although functional consequences remain to be explored.

However, what drives the selective enrichment of Firmicutes and Proteobacteria in *Ascaris-* and *Trichuris*-associated microbiomes, and why are Bacteroidetes bacteria commonly low? At present, no definitive answer has been established, and current explanations remain largely speculative. We found that this pattern is not limited to these parasites but has been demonstrated in other organisms, including free-living nematodes (e.g., *C. elegans*, *A. maximus*) (21, 22, 34, 35), trematodes (24, 36), and filarial parasites (18–20). One possible theory is that the environment within the parasites differs from the host guts, creating unique conditions depending on changes in nutrient availability, oxygen levels, and host immune response that favor bacteria to adapt and survive under stressful conditions. For example, Proteobacteria, particularly facultative anaerobes, can tolerate fluctuating oxygen levels and inflammatory conditions (37). In soil nematode *C. elegans*, Dirksen et al. highlighted that Proteobacteria contribute to nematode fitness, influencing development, population growth, and resistance to pathogens (22). Notably, certain *Pseudomonas* isolates demonstrated an *in vitro* anti-fungal effect, thereby enhancing the nematode’s defense against fungal pathogens. Other Proteobacteria members, *Ochrobactrum* and *Stenotrophomonas*, were also persistent under varying environmental conditions, demonstrating their adaptability in stressful conditions (22).

Another prominent phylum, Firmicutes, is well adapted to nutrient-limited and anaerobic conditions and has also been identified as dominant among free-living soil nematodes (34, 35). Chassard et al. provided insights into the capabilities of Firmicutes species, particularly the genus *Ruminococcus*, which can degrade complex polysaccharides and cellulose substrates (38). For instance, *Ruminococcus champanellensis* isolated from a human fecal sample exhibited the capacity to degrade microcrystalline cellulose. Moreover, Firmicutes play a role in modulating host immune responses, as demonstrated by Natividad et al., where germ-free mice colonized with ulcerative colitis microbiota showed higher sensitivity to colitis compared to mice colonized with microbiota rich in Firmicutes (39). This study identified bacterial families, such as Lachnospiraceae and Ruminococcaceae, as potential modulators of immune responses linked to colitis (39).

In contrast, Bacteroidetes are typically adapted to stable, nutrient-rich environments (40), which may limit their persistence in the internal environment of certain parasites such as *Ascaris*. Notably, a lower abundance of Bacteroidetes is more evident in *Ascaris* in studies involving natural human hosts (31, 32), compared to *Trichuris* (29) and soil nematodes such as *A. maximus* (21), suggesting that their presence is context dependent. Functionally, Bacteroidetes, such as *Bacteroides fragilis*, are involved in oxidative stress response, immunomodulation, and flavonoid metabolism (41, 42). We suggested that the reduced Bacteroidetes taxa in *Ascaris* may therefore be explained by variation in parasite biological characteristics and habitat in their hosts. *Ascaris* inhabits and dwells freely in the small intestine (has greater oxygen exposure, which is less favorable for strictly anaerobic bacteria) in contrast to *Trichuris*, which resides in the colon (more stable environment, anaerobic, and enriched in complex carbohydrates) (43). The *Trichuris* worm also embeds itself within the mucosal layer, potentially supporting more acquisition of Bacteroidetes in the *Trichuris* microbiota. Overall, these observations suggest that the selection of the parasite-associated microbiomes by horizontal acquisition is shaped by niche-specific ecological conditions and the host environment for better adaptation and survival ability to thrive in the hosts.

## POSSIBLE ROLES OF THE *ASCARIS-* AND *TRICHURIS*-ASSOCIATED MICROBIOMES

With emerging evidence indicating that the nematode holobionts of *Ascaris* and *Trichuris* have distinct and structured bacterial communities that are derived from, yet different from, their host’s gut microbiota, an important question arises: what roles do these bacteria play in parasite biology and host-parasite interactions? Experimental studies in *T. muris* strongly suggest that these associated bacteria are essential for nematode fitness and survival in the gut host, as their depletion via *ex vivo* antibiotic treatment leads to a significant decline in *T. muris* health and eventually death (29). In other nematodes, bacterial symbionts have been shown to be essential in helminth biology, including growth and fecundity (potential to produce offspring), as indicated in filarial parasites harboring *Wolbachia* (44). These microbes may also influence and shape crosstalk or interactions between nematode-host microbiota via physical contact and chemical signaling (31), facilitating stage progression and parasite establishment (33). This was demonstrated in *T. muris*, which exhibits a strong dependence on host microbiota for egg hatching, where even a single commensal host bacterium such as *Bacteroides thetaiotaomicron* can act as a surrogate to support parasite development and establishment (29), highlighting potential holobiont-holobiont interactions in the context of host-parasite relationships (26).

Beyond structural roles, parasite-associated microbiota may contribute to metabolic support and nutritional complementation. Klomkliew et al. reported enrichment of *Streptococcus* along with increased levels of metabolites such as amino acids, lipids, and nucleotide precursors within *A. lumbricoides* gut in heavy intensity of infection (31). This enrichment is linked to biosynthetic pathways for growth and reproduction, suggesting enhanced parasite fitness and egg production. In contrast, *Lactococcus* was found to predominate in worms recovered from hosts with low intensity of infection and was associated with carbohydrate metabolism, likely reflecting a survival state of the parasite rather than active proliferation (31). These findings suggest that parasite microbiota may contribute to disease progression, shifting from a relatively benign effect in low burdens of infections to a more pathogenic state in heavy infections.

It is important to distinguish when STH becomes detrimental, as low-intensity infections may exert immunomodulatory or beneficial effects, while chronic infections induce more pronounced microbial alterations and are associated with disease progression. Do parasite microbiomes shift in response to infection burden, and could such changes influence parasite physiology and host outcomes? In *T. muris*, the parasite is shown to manipulate the host gut microbiota, making the environment less favorable for egg hatching and further colonization (29). Complementary to this, Klomkliew et al. reported that the parasite microbiome in *A. lumbricoides* differed based on infection intensity, suggesting a potential “crowd control” mechanism to reduce competition and support prolonged establishment within the host (31).

Additionally, helminth-associated microbiota has been linked to modulating host immune responses, potentially complementing the already well-established ability of helminths to induce regulatory and anti-inflammatory pathways (13). Infection with *T. muris* was seen to induce a type-2 immune response characterized by increased IL-4 and IL-13 production from CD4+ T helper (Th2) cells, which activates STAT6 signaling in intestinal epithelial cells and stimulates goblet cell expansion as well as mucus secretion (15). This altered intestinal environment promotes the growth of *Clostridiales* while inhibiting colonization by pro-inflammatory bacteria such as *Bacteroides vulgatus* (15). *Wolbachia* endosymbionts in filarial nematodes, such as *Onchocerca* and *Dirofilaria*, produce essential metabolites, including riboflavin (vitamin B2), heme, glutathione, and glycolytic enzymes that support parasite development, and their removal through antibiotic treatment leads to delayed molting, reduced growth, embryonic failure, and eventual worm death (18, 45, 46). Furthermore, bacteria associated with *Trichuris* spp., including *Escherichia coli*, *Salmonella typhimurium*, and members of the *Peptostreptococcaceae* family, facilitate egg hatching by adhering to the polar plug of the whipworm egg using type-1 fimbriae. The bacteria then accumulate into dense, biofilm-like clusters, and the resulting physical and chemical signals trigger the larva to hatch (11, 14, 47). Gram-negative bacteria such as *Pseudomonas pyocyanea* (currently classified as *Pseudomonas aeruginosa*) have been reported to suppress gram-positive bacteria with anthelmintic activity, specifically *Bacillus mesentericus*, through antibiotic effects (14, 48). In the absence of *Pseudomonas*, *B. mesentericus* can cause lysis of the nematode tegument and death of *Ascaris*, indicating that bacterial interactions may indirectly protect the parasite (48). Moreover, infection with *A. lumbricoides* has been associated with alterations in host gut microbiota composition and abundance, highlighting the complex multilateral or tripartite interaction between host, parasite, and microbiota (31, 32). These alterations may create a more favorable environment for STH parasites and reduce inflammatory responses that could otherwise remove them from their infected hosts.

It is also interesting to note that some members of the *Ascaris-* and *Trichuris*-associated microbiota, such as *Bartonella*, *Mycobacterium*, *Rickettsia*, *Escherichia/Shigella*, and *Clostridium* (16, 30, 32), are known for their pathogenic potential in humans. This suggests that STH parasites may harbor and protect these pathogenic bacteria within their bodies, acting as reservoirs that facilitate their persistence and transmission between hosts (30). This is not surprising since their life cycle involves environmental stages in soil, which allow high exposure to diverse microbial communities. However, more confirmation studies using culture-based methods or whole-genome sequencing are needed, as 16S rRNA sequencing cannot distinguish between pathogenic and non-pathogenic strains or confirm active transmission.

Overall, rather than acting as passive microbial communities, current evidence supports the possible roles of STH microbiota in parasite development and fitness, metabolic functions, immune modulation, and disease progression. It is important to emphasize that most of the proposed functions of *Ascaris-* and *Trichuris*-associated microbiota are largely based on observational associations rather than direct validation that is waiting to be elucidated.

## LIMITATIONS, THEORETICAL CHALLENGES, RESEARCH GAPS, AND FUTURE DIRECTIONS

Despite the establishment of PMP in 2017 (27, 28), studies on STH-associated microbiomes remain limited, with several methodological discrepancies and theoretical limitations restricting current understanding. Most studies were largely descriptive, with some preliminary or based on small sample sizes, and often overlooked other biological aspects, including tissue-specific microbiota and transmission dynamics in natural settings. Firstly, most studies on the adult *Ascaris-* and *Trichuris-*associated microbiota have focused exclusively on worm gut tissue. Only one study has included a sample of reproductive tissue from *T. suis* (30) and reported enrichment of taxa such as *Enterococcus* in that tissue. However, no definitive conclusion could be made from this study due to the very limited sample size. This sampling bias may significantly constrain our ability to distinguish between the stable, core microbiota and the transient, environmentally acquired microbiota.

Current evidence, which is largely derived from the gut microbiota of adult worms, suggests a strong role of horizontal transmission (16, 29, 31–33), likely reflecting a dynamic and transient microbial composition influenced by host diet and gut environment. If we look further into the perspectives between the gut microbiota of male and female *A. lumbricoides* worms, no significant differences were observed in overall microbial structure (16), suggesting worm sex contributed minimally to microbiome variation. However, this perspective may be incomplete. More attention should be given to other tissues such as reproductive organs, including ovaries, which may harbor more stable microbial structures. By examining this compartment between male and female worms, we suggest the possibility of a sex-specific worm microbiome. Evidence from filarial nematodes supports this possibility, where endosymbiont *Wolbachia* is localized within reproductive tissues and plays a critical role in parasite fecundity, development, and pathophysiology (18, 19). Hence, we believe that STH parasites may have two types of microbiome systems: a horizontally acquired microbiota that supports adaptation and phenotypic variability (as mostly reported) and a core, perhaps vertically transmitted microbiota that contributes to reproductive success and parasite development, an aspect that needs to be unlocked.

Another limitation is the lack of studies characterizing the microbiota of human *A. lumbricoides* and *T. trichiura*. Most current knowledge was extrapolated from experimental models such as those of *A. suum* or *T. muris* (16, 29), or from natural infections in animals with *T. suis* and *T. trichiura* (30). Although these studies provide valuable insights, differences in host species, environmental, and experimental control settings may contribute to variation in bacterial signatures and limit direct translation to human infection. For instance, *in vitro* experimental studies on *T. muris* (29) and *A. suum* (16), the parasitic eggs and hatched larvae *in vitro* used were bacterium free or sterile. In contrast, under natural transmission, the embryonated eggs of *Ascaris* and *Trichuris* develop in soil environments rich in diverse soil microbial communities. This also raises a question: to what extent does the soil microbiome contribute to the initial acquisition and shaping of the parasite-associated microbiota during the early developmental stage in the soil? Integrating environmental microbiome analysis, particularly soil microbiome analysis in STH-endemic areas, may therefore provide insights into the ecological perspectives of parasite-associated microbiota. These gaps also indicate the need to adopt a One Health perspective (49), which recognizes the interconnectedness of human, animal, and environmental health. This concept has been expanded to integrate their microbiomes (50), highlighting that microbial communities may be shared and exchanged across these interconnected systems. Given the close evolutionary relationship between *A. suum* and *A. lumbricoides* (51, 52), as well as *T. trichiura*, *T. suum*, and *T. vulpis* (53, 54), and their shared environments in endemic regions, comparing microbiota across human and animal hosts and soil may also reveal both shared and host-specific microbial signatures. This approach is particularly relevant in STH-endemic populations where zoonotic transmission is common, as it may provide insights into transmission dynamics and facilitate the development of microbiome-based control strategies.

Another emerging area is the potential interaction between parasite-associated microbiota and their antimicrobial molecules. *A. suum* is known to produce antimicrobial peptides and excretory-secretory products, which may interfere with biofilm formation and the growth of bacteria (55). Although studies are limited, this finding may indicate that the parasite may selectively shape its microbiota through this antimicrobial activity by acquiring beneficial bacterial taxa and excluding the non-compatible bacteria.

In addition to the theoretical gaps, several other methodological challenges persist, as addressed previously (28, 44). These include the lack of standardized methodologies and the high risk of contamination in these nematode samples. Both papers emphasize the need for certain degrees of standardized protocols and the necessity of controls at every stage from sampling to sequencing to avoid contamination and ensure data reliability. Without such standardization, comparisons across studies remain challenging, hindering the ability to draw conclusions regarding *Ascaris-* and *Trichuris*-associated microbiomes. A key methodological advancement is to incorporate microscopy-based techniques such as fluorescence *in situ* hybridization or transmission electron microscopy (44) to localize parasite-associated microbiota within tissue, especially for potential members of the core microbiota.

Furthermore, most of the existing parasite microbiome studies are cross-sectional. Future studies should also focus on longitudinal studies, such as how the environmental factor of climate variation or host dietary pattern affects the parasite microbiome. In addition, the reliance on 16S rRNA sequencing in many studies may restrict the taxonomic profiling of microbiota and their functional potential. Advanced approaches such as shotgun metagenomics, metabolomics, and transcriptomics may provide better insight into these aspects.

Aside from improving our understanding of host-parasite interactions, studies on parasite-associated microbiota may provide new opportunities for the development of microbiome-based interventions to control STH infections. One promising strategy is to target bacterial communities that are essential for parasite survival or development. A well-established example is the use of the antibiotic doxycycline to target the intracellular endosymbiont *Wolbachia* in filarial nematodes, leading to parasite sterilization, impaired embryogenesis, and death of adult worms (20, 56, 57), while also reducing the severity of lymphedema in lymphatic filariasis (58). Although, to date, no obligate bacterial endosymbionts have been identified in STH parasites, depletion of parasite-associated bacteria impairs egg hatching, parasite development, and fitness (11, 29). Targeting these bacteria could complement existing anthelmintic (AHT) treatment, particularly as AHT resistance continues to emerge (59).

**Fig 1:**
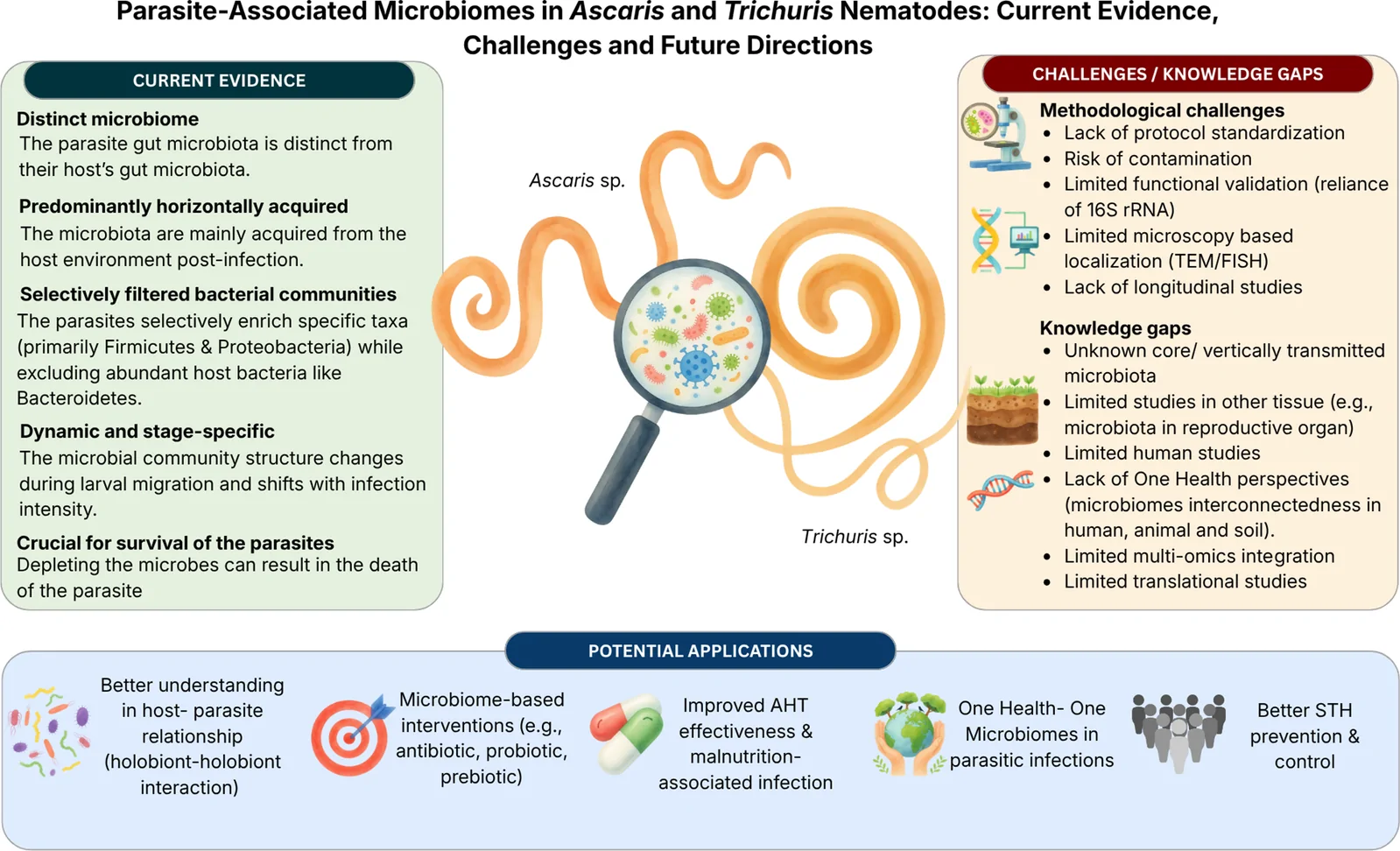
An overall summary of the current evidence, challenges, major knowledge gaps, and potential applications of parasite-associated microbiota in STH parasites.

Another potential approach is to manipulate the host gut microbiota through probiotics, prebiotics, dietary interventions, or microbiome-derived metabolites (e.g., short-chain fatty acids) (60–62). These strategies have been proposed to reduce parasite burden and establishment by creating a less favorable gut environments through modulation of the host gut microbial composition, enhancement of the intestinal barrier, and regulation of host immune responses (60–62). Experimental animal studies have also shown that probiotic strains of *Lactobacillus* and *Bifidobacterium* can modulate host immune responses and alter the gut microbial environment following *T. muris* and *A. suum* infections (63, 64). Additionally, microbiota-based interventions could also complement deworming programs by potentially enhancing the effectiveness of AHT treatment while simultaneously improving infection-associated malnutrition in STH-endemic populations (65). An overall summary of the current evidence, challenges, major knowledge gaps, and potential applications of parasite-associated microbiota in STH parasites is presented in Fig. 1.

## CONCLUSION

Collectively, research on *Ascaris-* and *Trichuris*-associated microbiota for the understanding of host-parasite interactions in STH infections is still in its infancy, with many arising questions yet to be addressed. Nevertheless, available evidence consistently supports the nematodes to harbor structured, non-random bacterial communities that are derived but distinct from those of their host's gut microbiota, depending on parasite niche, life stage, and host environment. Although direct validation is lacking, these parasite-associated microbiota could be associated with parasite development, fitness, and survival, as well as manipulation of the host microbiota, highlighting their role as important components of the parasite holobiont. While current studies largely support the dominance of horizontally acquired and host-derived microbiota, important gaps remain in identifying potential core microbial communities. Ultimately, this knowledge is not just important for our understanding of the biology of STH parasites but also supports the development of innovative, microbiome-based interventions to control STH infections in endemic settings.
